# Healthcare professionals’ perceptions and experiences of obesity and overweight and its management in primary care settings: a qualitative systematic review

**DOI:** 10.1017/S1463423623000683

**Published:** 2024-01-17

**Authors:** Laura Jeffers, Jillian Manner, Ruth Jepson, John McAteer

**Affiliations:** Scottish Collaboration for Public Health Research and Policy, School of Health in Social Science, University of Edinburgh, Edinburgh, UK

**Keywords:** healthcare professionals, obesity, qualitative research, stigma, systematic review, therapeutic relationship

## Abstract

**Aim:**

This qualitative systematic review aimed to synthesise existing qualitative research on HCPs’ perceptions and experiences of obesity and its management in primary care settings.

**Background:**

Healthcare professionals (HCPs), particularly those in primary care, play a key role in policy implementation around weight management. Overweight and obese individuals are subject to weight stigma which has negative health consequences and reduces the likelihood of healthcare service usage. An understanding of HCPs’ perceptions of obesity and weight management in primary care is necessary for the development and delivery of effective initiatives.

**Methods:**

A search strategy developed using the SPIDER framework was applied to Medline and CINAHL databases. Inclusion criteria were applied, and quality assessment was undertaken using the CASP framework. Fifteen papers meeting the inclusion criteria were analysed thematically.

**Findings:**

Four themes were identified: conflicting discourses surrounding obesity, medicalisation of obesity, organisational factors, and lack of patient knowledge and motivation. Conflicting discourses around obesity refers to the differing views of HCPs regarding what it means to have and treat obesity. Medicalisation of obesity considers whether obesity should be treated as a medical condition. Organisational factors were identified as knowledge, resources and time that affected HCPs’ ability to provide care to overweight or obese. Finally, the review discovered that patients required their own knowledge and motivation to lose weight. This review has highlighted the need to provide safe, non-judgemental spaces for HCPs and patients to discuss weight and weight loss. This is essential to the therapeutic relationship and the provision of effective obesity management.

## Background

Overweight or obese individuals are at an increased risk of cardiovascular disease, type two diabetes and musculoskeletal disorders (World Health Organization, 2021). For the purpose of this research, the terms overweight and obesity have both been considered. Overweight refers to individuals with a body mass index (BMI) greater or equal to 25, and obese refers to individuals with a BMI greater or equal to 30 (WHO, 2021). Elevated BMI has negative effects on mental health and is linked to depression and anxiety (Luppino *et al*., 2010). In 2016, approximately 39% of adults in the world were overweight or obese (WHO, 2021). The cost to UK health services of overweight individuals is between £363 and 600 million, with many of these costs linked to co-morbidities associated with obesity (Castle, 2015). Tackling obesity is therefore a priority for governments worldwide. In high-income countries such as England, 36% of adults living in the most deprived neighbourhoods suffer from obesity in comparison to 20% of adults living in the least deprived neighbourhoods (Adams, 2020), illustrating how socio-economic factors affect weight.

As the health service’s front-line defence against obesity, healthcare professionals (HCPs) in primary care are in the optimum position to deliver weight management care. Primary care practitioners can understand the unique health disparities as well as cultural and social landscapes of the communities they serve. It is paramount to understand HCPs’ perceptions of overweight and obesity and weight management in primary care to aid the delivery of effective initiatives while accounting for health inequalities. For example, the UK National Health Service (NHS) Long Term Plan sets out how the service will move towards a preventative model of healthcare where practitioners and patients share responsibility for health (NHS, 2019). However, said plan does not outline how a preventative model will be achieved, hence the need for further research into healthcare issues that can be prevented, such as obesity.

The relationship between individuals and their healthcare provider is key to discussing sensitive topics such as weight and in ensuring that care is person-centred (Dempsey *et al*., 2016; Greenhalgh and Heath, 2010). A good therapeutic relationship is one in which patients trust their clinician, perceive them to be empathetic and feel comfortable enough to share concerns (Carey *et al*., 2012). This leads to patient and clinician satisfaction and positively impacts treatment compliance (Greenhalgh and Heath, 2010). It is important to explore how health professionals perceive their patients and the health issues that they present with (e.g., being overweight), since this can impact upon the therapeutic relationship and the extent to which the patient engages with appropriate healthcare.

Weight stigma is a well-documented issue which refers to negative attitudes and beliefs held against or negative stereotypes of overweight individuals, often resulting in discrimination (Ogden, 2014). Weight stigma is present across education, healthcare and media sectors and can be expressed by family and friends (Levy and Pilver, 2012). This can lead to internalised stigma, resulting in negative self-evaluations, depression, anxiety, body dissatisfaction and poor self-esteem, in addition to being positively associated with diabetes risk and high cortisol levels (Durso and Latner, 2008; Wu and Berry, 2017). Evidence suggests that an HCP’s conscious or unconscious weight stigma can impact the care provided to an overweight patient. HCPs in maternity care showed negative attitudes towards caring for overweight and obese patients, and they were perceived to have poorer self-management behaviours (Mulherin *et al*., 2016). HCPs in primary care were reported to inappropriately focus on weight during an appointment (Phelan *et al*., 2015). Consequently, overweight and obese individuals are more likely to delay accessing medical services as they believe they will not receive appropriate care or be disrespected because of their weight (Amy *et al*., 2005). This increases the likelihood of disengagement with healthcare services, resulting in poorer health outcomes (Phelan *et al*., 2015). Therefore, it is clear that societal weight norms can impact the care HCPs provide. This further illustrates the need to investigate HCPs’ perceptions and their effect on obesity management.

## Aims

The aim of this review is to synthesise the qualitative research on HCPs’ perceptions and experiences of obesity and its management in primary care settings.

The objectives are to:

- Understand the perceptions of HCPs towards overweight and obesity and weight management.
- Understand the experiences of developing and maintaining a therapeutic relationship when discussing weight and weight management with patients.
- Understand the resources (e.g., education) needed to develop a therapeutic relationship with patients who are overweight or obese.

## Methods

**Figure 1.**
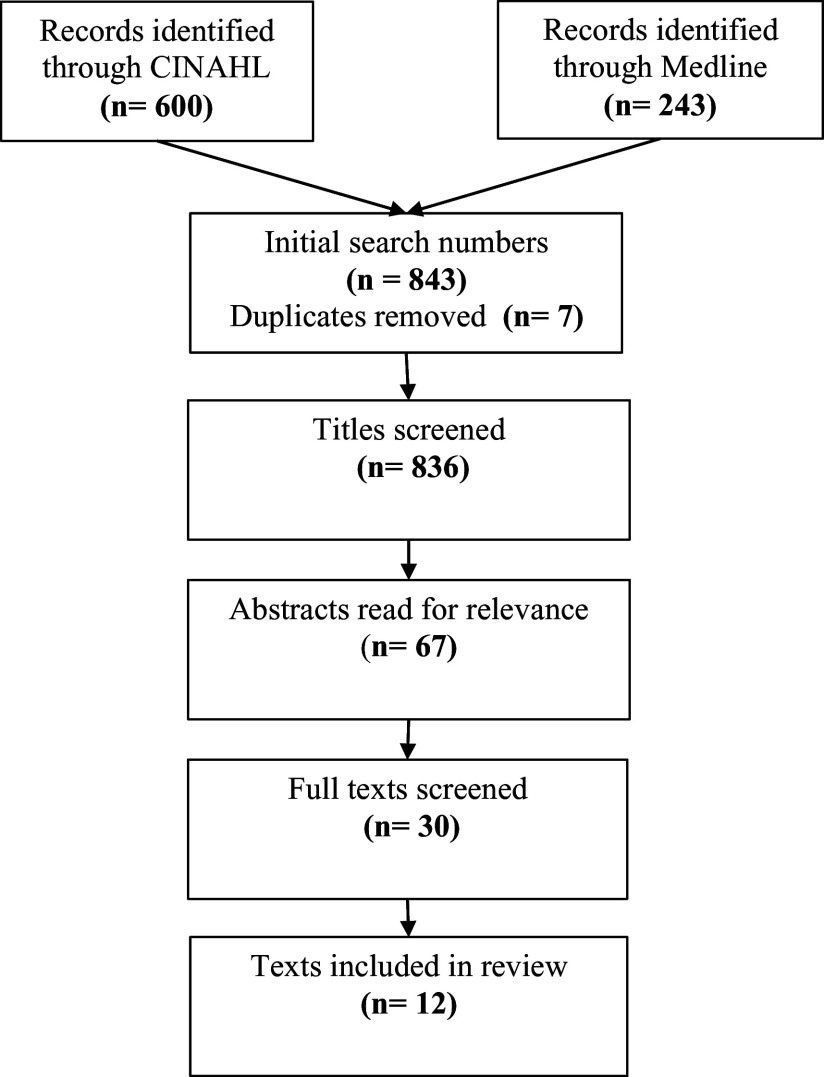
PRISMA diagram January 2021

**Figure 2.**
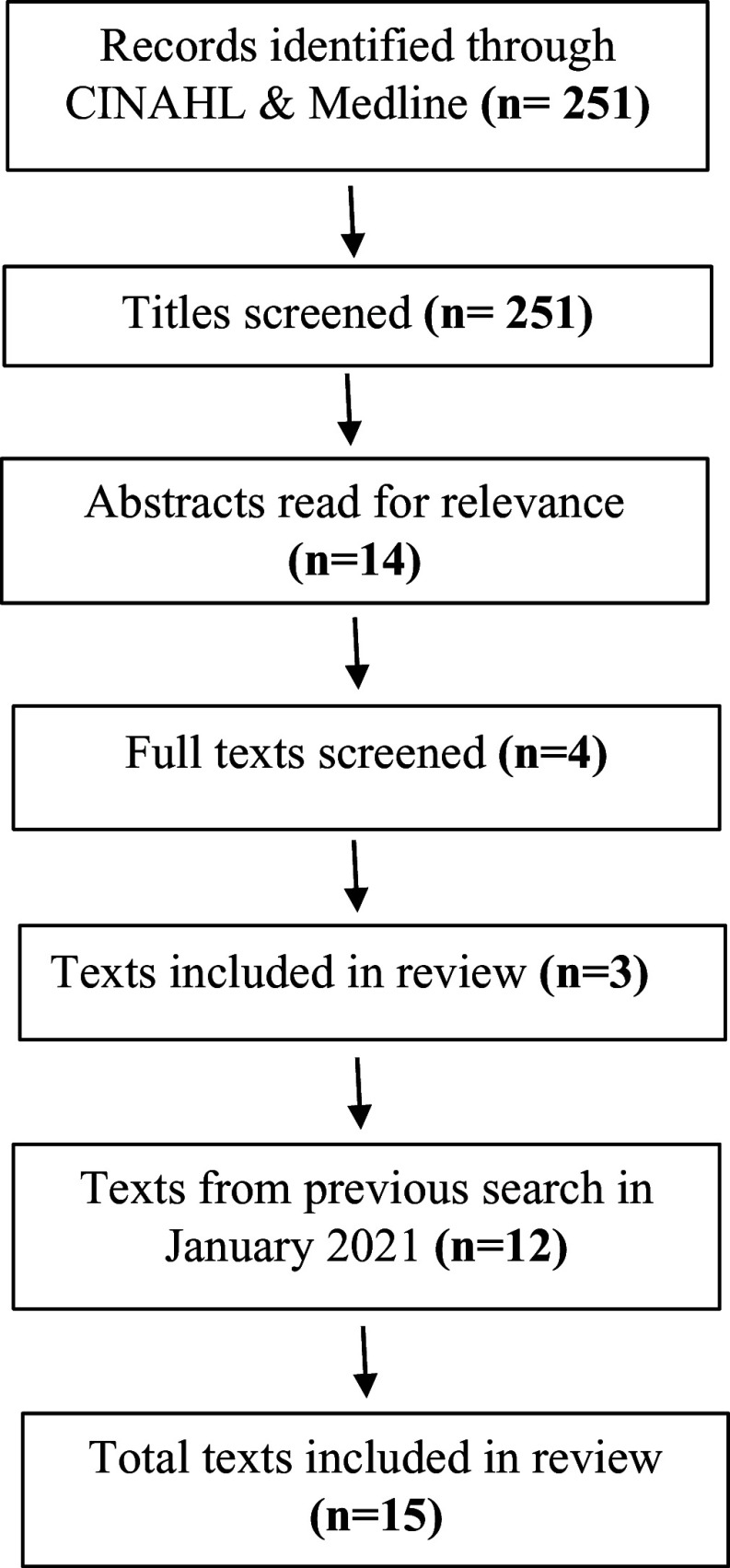
PRISMA diagram October 2023

A systematic review was conducted in November 2021. The search was updated in October 2023. The steps of the review and updated review are shown in Figure 1 and Figure 2 respectively.

### Inclusion criteria and populations and phenomenon of interest

The inclusion criteria were developed using the SPIDER (Sample, Phenomenon of Interest, Design, Evaluation, Research type) framework as seen in Table 1 (Cooke *et al*., 2012). Analysis of patient perceptions was not within the scope of this paper. However, papers reporting perceptions of HCPs alongside those of patients were included if these were reported separately. Papers reporting perceptions related to the delivery of a specific intervention were excluded, since these findings were considered only generalisable to the specific intervention being studied.

**Table 1.** Application of SPIDER framework (Cooke *et al.*, 2012)

Sample	The sample being researched in this case is healthcare professionals.
Phenomenon of interest	The phenomenon of interest is the provision of weight management services to those who are overweight or obese. The phenomenon of interest also extends to understanding the effects of providing weight management services on the therapeutic relationship.
Design of research	Focus groups or interviews where semi-structured or open questions were asked rather than quantitative methods or surveys as this is beneficial in gaining full understanding of the perceptions and attitudes of the sample population.
Evaluation	The experiences, perceptions and attitudes of healthcare professionals will be examined.
Research type	Qualitative research only as this was most appropriate for an in-depth exploration of the perceptions, attitudes and experiences of healthcare professionals.

Only papers reporting qualitative findings using interviews or focus group methodologies were included as these were deemed most appropriate for analysing perceptions and attitudes of the sample population (Moule *et al*., 2016). Qualitative papers using surveys were excluded as surveys often do not gain adequate depth of understanding from participants compared to focus groups or interviews (Ring *et al*., 2011; Jones, 2013). Papers involving weight management of children were excluded as there is no relevance to adult nursing. The setting also excluded multiple papers due to being set in secondary care.

### Delimiters

It was required that papers were peer-reviewed and in English. No year limiters were set as there was no reason to exclude papers published at an earlier date. However, all papers were published in the last 20 years.

### Search strategy

A search strategy was developed by three researchers using the SPIDER framework as seen in Table 1. The SPIDER framework was chosen due to the qualitative nature of the research question. The SPIDER framework uses the same principals as the well-known PICO (Problem, Intervention, Comparison/Control, Outcome) framework but applies it to qualitative and mixed-method studies (Cooke *et al*., 2012). Search terms were agreed, and a search string applied to both CINAHL and Medline, as shown in Table 2.

**Table 2.** Search String applied to CINAHL and Medline

1. ‘nurs*’ OR ‘district nurs*’ OR ‘school nurs*’ or ‘family nurs*’
2. ‘primary care’ OR ‘communit*’ or ‘general practice’ or ‘GP’ or ‘health cent*’ OR ‘medical cent*’ OR ‘family practice’
3. ‘obese’ OR ‘obesity’ OR ‘overweight’ OR ‘BMI’ or ‘body mass index’ OR ‘body fat’ OR ‘bariatric’ OR ‘overweight’ OR ‘unhealthy weight’ OR ‘fat”
4. ‘qualitative research’ OR ‘qualitative stud*’ OR ‘qualitative method*’ OR ‘interview*’ OR ‘focus group*’
5. 1 and 2 and 3 and 4

### Applying the inclusion criteria

Inclusion criteria were applied to titles, abstracts and full paper. A second researcher screened 10% of titles, abstracts and full papers generated by the search. Eighty-three titles were double-screened. Reviewers agreed on 76 (92% agreement) and disagreed on 7. Seven abstracts were double-screened. Reviewers agreed on six (86% agreement). Three papers were double-screened with agreement being reached on all (100% agreement). Disagreements were resolved through discussion. The levels of agreement at each stage were high which indicates an appropriate level of agreement.

### Quality assessment

The CASP Qualitative Papers Checklist was used to assess the quality of included papers (CASP, 2018) (Table 3). This tool was chosen to reduce the subjectiveness related to the appraisal while covering all areas needed to critically appraise the evidence (Nadelson and Nadelson, 2014). However, due to the qualitative nature of the evidence some subjectiveness may remain. Overall, the quality of evidence was good with CASP scores ranging from 7 to 10. All papers appropriately used qualitative methodology and illustrated rigorous data analysis methods. The main criterion on which papers scored poorly was the specification and exploration of relationships between researchers and participants. This makes it challenging to understand how researchers may have influenced results in terms of possible biases or power differences between themselves and participants (Holloway and Galvin, 2016). The final question of the CASP checklist ‘how valuable was the research?’ was graded by assessing whether the research paper acknowledges how their research contributes to current research, practice and policy, whether the paper identified any new areas for research, and whether the researcher considered how the research may be beneficial to other populations. If these factors were acknowledged, the research was considered valuable and graded as ‘yes’.

**Table 3.** CASP quality assessment checklist

Author/title	Was there a clear statement of the aims?	Is a qualitative methodology appropriate?	Was the research design appropriate to address the aims of the research?	Was the recruitment strategy appropriate to the aims of the research?	Was the data collected in a way that addressed the research issue?	Has the relationship between researcher and participants been adequately considered?	Have ethical issues been taken into consideration?	Was the data analysis sufficiently rigorous?	Is there a clear statement of findings?	How valuable is the research?	Total
Ali *et al*., 2008	NO	YES	Can’t tell	YES	YES	YES	NO	YES	YES	YES	7
Blackburn *et al*., 2015	YES	YES	YES	YES	YES	NO	Can’t tell	YES	YES	YES	8
Bornhoeft, 2018	YES	YES	YES	YES	YES	NO	YES	YES	YES	YES	9
Brown and Thompson, 2007	YES	YES	YES	NO	YES	Can’t tell	YES	YES	YES	YES	8
Dunkley *et al*., 2009.	YES	YES	Can’t tell	NO	YES	Can’t tell	YES	YES	YES	YES	7
Gunther *et al*., 2012.	YES	YES	YES	YES	YES	YES	YES	YES	YES	YES	10
Hayes *et al*., 2017	YES	YES	YES	YES	YES	Can’t tell	YES	YES	YES	YES	9
Holmgren *et al*., 2019	YES	YES	YES	NO	YES	YES	YES	YES	YES	YES	10
Kirk *et al*., 2014	YES	YES	YES	YES	YES	YES	YES	NO	YES	YES	9
Mercer & Tessier, 2001	YES	YES	YES	NO	NO	YES	NO	YES	YES	YES	7
Phillips *et al.*, 2014	YES	YES	NO	YES	YES	Can’t tell	YES	YES	YES	YES	8
Rand *et al*., 2017	YES	YES	YES	YES	YES	Can’t tell	YES	YES	YES	YES	9
Abouied *et al*., 2022	YES	YES	YES	YES	YES	NO	YES	YES	NO	YES	8
Norman *et al*., 2023	YES	YES	YES	YES	YES	NO	YES	YES	YES	YES	10
Brautigam Ewe *et al*., 2021	YES	YES	YES	YES	YES	YES	NO	YES	YES	YES	9

### Analysis

Thematic analysis was used to identify themes for discussion. The results section of each paper was coded line by line, and initial themes developed. The initial themes were further refined to identify the best fit, and name for each theme and ensure thorough analysis of the research conducted (Braun and Clarke, 2012; Aveyard, 2019). One paper was double-coded by two of the authors to ensure coding related to the study aims and to consider different perspectives. The first author then developed an iterative list of themes which captured the relevant themes based on the first coded paper. The remaining papers were coded by the first author based on this initial list, and new codes were added as new and relevant themes were identified in subsequent papers. The exact themes and subthemes were refined through discussion with the second and third author until no new themes were identified.

## Results

### Characteristics of included studies

In total, 15 papers were included in the review. A summary of results can be seen in Table 4. Six papers were conducted in the United Kingdom, two in the United States, three in Canada, one in the United Arab Emirates (UAE), two in Sweden and one in New Zealand illustrating the cultural range of the research included. Five papers reported data from nurses alone, with the remaining papers reporting data from numerous community HCPs. The most common method of recruitment was purposive sampling. Papers had a sample size of between 10 and 33. The main method of data collection was interviews (*n* = 14). One paper also used a combination of focus groups and interviews. Thematic analysis was the most common method of analysis (*n* = 11). One paper used a combination of grounded theory and thematic analysis and one used both discourse and thematic analysis.

**Table 4.** Results summary

Author, title and setting	Aim	Sample/design	Results/themes identified
Ali *et al*., 2008. United Arab Emirates.	Qualitative paper to explore weight management barriers for Emirati women and strategies that can facilitate their weight management efforts from a healthcare professionals’ perspective.	29 participants. Semi-structured interviews. Grounded theory was used to guide data collection and analysis. Thematic analysis.	Barriers to weight management fell into four categories: Personal Healthcare system Community Policy
Blackburn *et al*., 2015. United Kingdom, England.	To explore GPs and primary care nurses’ perceived barriers to raising the topic of weight in general practice.	Purposive sampling. 17 nurses and 17 GPs. Theoretical domains framework. Content analysis.	Four themes were identified: Limited understanding about obesity care. Concern about negative consequences and fear of potential damage to the patient relationship. Lack of time and resources to raise a sensitive topic.
Bornhoeft, K. 2018 United States	To develop an understanding of the perceptions, attitudes and beliefs held by primary care providers (PCPs) on the subject of obesity in order to shed light on the barriers preventing effective obesity management.	Purposive sampling. 12 participants. Semi-structured interviews Thematic analysis.	Three themes were identified: Provider-centered obstacles. Organisational obstacles separated into subthemes: organisational culture and support and services. Provider perception and patients’ obstacles.
Brown & Thompson, 2007. United Kingdom, England	To explore primary care nurses’ attitudes, beliefs and perceptions of own body size in relation to giving patients advice about obesity.	Postal survey recruitment. 33 participants, 100% female. Face-to-face interviews. Thematic analysis.	Three themes were identified: Sensitivity about obesity. Complexity of obesity. Own body size.
Dunkley *et al*., 2009. United Kingdom, England	To determine the knowledge and attitudes of patients and primary care practitioners concerning waist circumference measurement (WCM), with particular reference to exploring barriers in a multi-ethnic setting.	Purposive sampling. 10 HCPs from 9 GP practices. Face-to-face interviews. Open coding was used for the first 3 interviews. A coding guide was developed and then used to code the remainder of the interviews.	HCPs lack understanding of the link between a large waist size and risk of health conditions such as diabetes and CVD. HCPs expressed that they required standardised guidelines to implement waist measurement; however, they did feel it was more useful than BMI. HCPs were concerned about the intimate nature of taking waist circumference measurements and expressed that lack of time was a barrier to carrying this out.
Gunther *et al*., 2012. East Midlands, United Kingdom.	To uncover and describe barriers and enablers to implementing NICE’s recommendations on the management of obesity in adults and general practice, using practical qualitative methods.	Purposive sampling. 14 HCPs from 7 GP practices. Face-to-face semi-structured interviews. Thematic analysis.	Reluctance to take responsibility for implementing a weight guideline. Lack of consistency in weight management approaches. Issues relating to patient consultations such as lack of practitioner confidence or lack of services to offer.
Hayes *et al*., 2017 United States	To identify the challenges of primary care professionals in regard to the management of obesity.	31 participants. Participants were interviewed over the telephone and also participated in a virtual focus group. Each focus group lasted 75 minutes and interviews 45 minutes. Thematic analysis.	Three themes were identified: Inconsistent primary care team integrations. Challenge’s conceptualising obesity as a chronic condition. Acknowledgement that HCPs lack of knowledge, frustration, bias and prejudice could be perceived by patients.
Holmgren *et al*., 2019 Sweden	To develop a theory to explain how PHN (Public Health Nurses) accomplish and adapt counselling in lifestyle habits to decrease obesity in people with MD (Mobility Disability). Research questions were guiding the paper. 1. What is the main concern when guiding people with MD in the lifestyle change counselling and treatment to decrease obesity, and 2. How to PHNs solve this concern?	Classic grounded theory with face-to-face interviews. 10 nurses with a master’s degree in public health. The first three interviews were open-coded and used to develop guidance for the following interviews. Data being collected were continually analysed. Once the core concept was developed, the questions were developed and placed within the rest of the interviews. The tenth interview brought no new data.	Barriers and enablers were identified to initiating a conversation about weight. These included: Person centredness in the situation. Experience and knowledge. Strengthening conditions, for example, motivation. Access to other professionals. Prioritisation of everyday work.
Kirk *et al*., 2014 Canada	To examine the experiences of individuals living with obesity, the perceptions of healthcare providers and the role of social, institutional, and political structures in the management of obesity.	Feminist post-structuralism. Purposive sampling. 16 HCPs. 8 dieticians, 4 nurses and 4 physicians. Face-to-face interviews. Discourse and thematic analysis.	Blame for being overweight was seen across all groups. HCPs also blamed themselves for not having the correct information. Tensions in obesity management and prevention. Evidence of the prevailing medical management discourse.
Mercer & Tessier, 2001 Scotland, United Kingdom.	To examine GPs and PNs perceptions of obesity, their strategies and attitudes towards weight management, and their views on the major obstacles to (and need for) better weight management in primary care.	20 participants, 10 GPs and 10 PNs. Face-to-face interviews. Thematic analysis.	Identified a number of themes and issues: Both GPs and PNs felt it was not their role to address weight. Lack of routine management of overweight patients. PNs and GPs identified the need to promote long-term lifestyle changes. There was a positive view towards commercial slimming clubs. HCPs were more open to addressing weight if it was in the context of associated disease. HCPs saw more women than men regarding weight management. Weight management guidelines were not commonly used. All HCPs were against the use of weight-modifying drugs. All HCPs identified they required more support in terms of improving weight management. Identified a wide range of influences on weight at the family and societal level.
Phillips *et al*., 2014 United Kingdom, Wales	To use qualitative semi-structured interviews to explore how practice nurses manage obesity within primary care and to identify good practice and explore barriers to achieving effective management.	Purposive sampling. 18 semi-structured interviews. A body morph diagram was used in order to facilitate discussion surrounding which patients’ nurses would approach. Thematic analysis. Primary paper to Rand *et al*., 2017.	HCPs saw a range of patients for weight, and these patients were generally those suffering from chronic diseases. Nurses sometimes did not approach the topic of weight due to time constraints or fear or stigmatising the patient. Nurses were unclear whether they should approach overweight patients who were healthy. Nurses felt that building a relationship with the patient was key to approaching the topic of weight. They noted that many patients wanted quick fixes, but nurses resorted to giving encouragement and general weight and diet advice.
Rand *et al*., 2017. Canada	How do those living with obesity describe their experiences with psychological, emotional and social issues? What support do those living with obesity feel they need to promote positive mental wellbeing? What psychological, emotional and social issues do HCPs perceive are problems for their patients living with obesity? What supports do healthcare professionals require to promote the positive mental wellbeing of their patients?	19 individuals with obesity and 16 primary HCPs (eight dieticians, four family physicians and four nurses). Secondary paper, primary paper = Phillips *et al*., 2014. Two theoretical frameworks were selected: 1. a mental wellbeing framework and 2. socio-ecological model (SEM) was applied to categorise the mental wellbeing themes identified. Secondary theoretical thematic analysis.	Individual – HCPs agreed that food behaviours are addictive and arose from a symptoms of mental wellbeing issues. Interpersonal – HCPs expressed that they had seen condemnation of those with obesity by other HCPs. Organisational – multiple HCPs recognised a missing component of obesity management was addressing mental wellbeing. Community – most HCPs did not acknowledge the broader social stigma attached to obesity in the community. Policy – HCPs made very little reference to policies.
Aboueid *et al*., 2022 Canada	This study aimed to investigate how health professionals communicate about weight with their patients.	Descriptive case study approach. Semi-structured interviews. 33 healthcare professionals. Thematic analysis.	Four themes identified: Analysing patient perspectives. Focusing on overall health rather than weight. Directly addressing the topic. Avoiding the topic.
Norman *et al*., 2023 New Zealand	The aim of this study was to explore the rural nurse practitioner experience with barriers to delivering weight management healthcare in their practice with a view to identifying areas of healthcare improvement.	Case study design. Semi-structured interviews. 10 rural nurses. Deductive analysis approach.	Three themes identified: Limitations of a nurse role. Patient-level barriers Cultural barriers.
Brautigam Ewe *et al*., 2021 Sweden	To describe primary care nurses’ experiences of patients being overweight or obese, as well as primary care nurses’ perceptions of overweight problems in society and visions working with lifestyle issues.	Descriptive design with a qualitative method and inductive approach. Semi-structured face-to-face interviews. 13 practice nurses. Qualitative content analysis.	The analysis of interviews resulted in three categories and nine sub-categories: Primary care nurses wish to promote health and prevent illness. Arenas for health promotion in society. Support patients to change their behaviour. Sub-categories: Schools and media’s impact on lifestyle. Regulation of the food market. Health promotion outside of the healthcare mission. A good patient meeting creates the conditions for lifestyle change. Lack of knowledge how to implement guidelines. Ethical and cultural issues challenge the nurse’s role. Tailored interventions as a meeting point for patients. The patient’s motivation determines the outcome. The individual has to choose a healthy lifestyle.

### Themes

Four themes were identified: (i) conflicting discourses surrounding obesity; (ii) medicalisation of obesity; (iii) organisational factors and (iv) lack of patient knowledge and motivation. Three subthemes were identified in relation the theme iii. A summary of themes and subthemes is seen below (Table 5).

**Table 5.** Summary of themes identified

Theme	Summary	Studies that illustrated theme
**i) Conflicting Discourses Surrounding Obesity**	Conflicting discourses were illustrated by efforts to reduce stigma towards overweight patients contrasted with ideas that actively enforced this stigma. Additionally, some HCPs also illustrated normalisation of larger body sizes and the effect of their own body size in relation to giving weight advice.	Ali *et al*., 2008 Blackburn *et al*., 2015 Bornhoeft, 2018 Brown & Thompson, 2007 Dunkley *et al*., 2009 Hayes *et al*., 2017 Kirk *et al*., 2014 Mercer & Tessier, 2001 Phillips *et al*., 2014 Rand *et al*., 2017 Norman *et al*., 2023 Brautigam Ewe *et al*., 2021 Aboueid *et al*., 2022
**ii) Medicalisation of Obesity**	Illustrates arguments for and against the medicalisation of obesity displayed by HCPs.	Blackburn *et al*., 2015 Brown & Thompson, 2007 Hayes *et al*., 2017 Kirk *et al*., 2014 Phillips *et al*., 2014 Brautigam Ewe *et al*., 2021 Norman *et al*., 2023 Aboueid *et al*., 2022
**iii) Organisational Factors** *Subthemes:* *Knowledge* *Resources* *Time*	The idea that organisations do not place enough focus on obesity management. This was illustrated by lack of knowledge, resources and time. *Knowledge –* HCPs did not have sufficient knowledge to give patients, nor were they aware of referral options available. *Resources –* HCPs explained that there were not enough resources to offer patients in terms of information and referral options. *Time –* HCPs did not have enough time to raise the topic of weight in a consultation.	Ali *et al*., 2008 Blackburn *et al*., 2015 Bornhoeft, 2018 Brown & Thompson, 2007 Dunkley *et al*., 2009 Gunther *et al*., 2012 Hayes *et al*., 2017 Holmgren *et al*., 2019 Kirk *et al*., 2014 Mercer & Tessier, 2001 Rand *et al*., 2017 Brautigam Ewe *et al*., 2021 Norman *et al*., 2023
**iv) Lack of Patient Knowledge and Motivation**	HCPs felt that the patient’s own knowledge and motivation were key to their ability to successfully lose weight.	Ali *et al*., 2008 Bornhoeft, 2018 Brown & Thompson, 2007 Holmgren *et al*., 2019 Mercer & Tessier, 2001 Phillips *et al*., 2014 Rand *et al*., 2017 Brautigam Ewe *et al*., 2021 Norman *et al*., 2023 Aboueid *et al*., 2022

#### Theme 1: Conflicting discourses surrounding obesity

Twelve papers reported conflicting ideas as to whether wider social, cultural and psychological factors in addition to physical factors affect weight. Whilst some HCPs acknowledged that overweight individuals face stigma within society, others actively enforced this stigma. HCPs acknowledged that their own weight played a part in giving weight advice to others. There was also evidence that some HCPs downplay the severity of obesity, referring to the ‘plus-size movement’ which aims to normalise and reduce stigmas associated with larger body sizes (Muttarak, 2018). This is likely to result in a variety of care outcomes for patients. HCPs who actively enforce stigma risk compromising the therapeutic relationship and thus engagement in care whilst HCPs who normalise being overweight risk contributing to a patient’s poorer health outcomes.

A number of factors were identified by HCPs as affecting patient’s ability to manage weight. Some studies reported that HCPs perceived weight loss as simply being down to eating less and doing more (Brown and Thompson, 2007; Kirk *et al*., 2014). One HCP said ‘*it will work if you just stick with it’* referring to losing weight by simply following a healthy diet (Kirk *et al*., 2014). In contrast, other papers described HCP perceptions of weight loss as being broader and incorporating a range of factors. For example, in two papers HCPs described obesity as a wider societal issue saying society is ‘*weight obsessed’* (Kirk *et al*., 2014), and a *‘society shift*’ was required for patients to lose weight (Mercer and Tessier, 2001). Other HCPs illustrated potential social and cultural factors contributing to weight gain explaining that patients’ families ‘*like’* them when they are overweight compared to when they are not (Bornhoeft, 2018). In a paper conducted in the UAE, HCPs mentioned cultural factors such as having large, shared meals and ‘*families not allowing women to go to fitness clubs’* as reducing women’s ability to lose weight (Ali *et al*., 2008). Lastly, psychological factors were acknowledged to play a part, with HCPs in three papers arguing that consideration of mental health in weight loss management was ‘*missing’* (Rand *et al*., 2017) and being overweight was *‘loaded mentally’* (Brautigam Ewe *et al*., 2021; Norman *et al*., 2023).

Weight stigma was also acknowledged by HCPs. Five papers reported HCP awareness of weight stigma, with participants stating that they are conscious of this when having conversations around the topic of weight (Brown and Thompson, 2007; Bornhoeft, 2018; Hayes *et al*., 2017., Rand *et al*., 2017; Norman *et al*., 2023). HCPs said they did not want to ‘*judge*’ (Brown and Thompson, 2007) or ‘*offend*’ patients (Rand *et al*., 2017) as this would compromise the therapeutic relationship. The fact that overweight patients may be ‘*perceived differently’* and in a ‘*negative*’ way was also taken into consideration by HCPs (Brown and Thompson, 2007). Conversely, three HCPs actively enforced stigmatisation, calling patients ‘*lazy*’ (Bornhoeft, 2018; Rand *et al*., 2017) and less likely to ‘*care’* about their health (Bornhoeft, 2018; Hayes *et al*., 2017). In contrast to weight stigma, there was some evidence of HCPs normalising being overweight, describing it as the ‘*the new normal’* (Bornhoeft, 2018). Other HCPs downplayed the consequences of being overweight saying they were aware of overweight people who were ‘*fit as fleas’* and had ‘*no problems’* (Dunkley *et al*., 2009). Another HCP stated they felt weight measurement guidelines were ‘*slightly extreme’* (Phillips *et al*., 2014).

HCPs also acknowledged their own weight when managing patients who were overweight. In four papers, HCPs with a higher BMI felt they were not able to give weight advice or felt hypocritical, saying they would be a *‘better role model*’ if they were thinner (Brown and Thompson, 2007; Blackburn *et al*., 2015; Bornhoeft, 2018; Aboueid *et al*., 2022). Weight stigma also applied to overweight HCPs saying ‘*everyone’s judging each other*’ implying there was reciprocal judgement between both patient and HCP (Bornhoeft, 2018). One HCP said they felt patients’ *‘focus’* on her stomach when having conversations surrounding weight. By contrast, being overweight was sometimes seen as a facilitator to conversations about weight as HCPs with a higher BMI were in a better position to empathise with overweight patients (Brown and Thompson, 2007). Some HCPs with higher BMI stated that they could provide advice based on experience saying they ‘*pass on tips*’ (Brown and Thompson, 2007). Two HCPs with lower BMI also expressed feeling ‘*preachy*’ and patients thought they did not understand what it was like to be overweight (Blackburn *et al*., 2015; Brautigam Ewe *et al*., 2021). To avoid this, some HCPs discussed weight loss experiences of friends and presented themselves as weak in another area of health such as exercise (Brown and Thompson, 2007).

In one paper, HCPs were able to acknowledge the socio-economic factors that affected their patients (Norman *et al*., 2023). HCPs in this paper acknowledged that patient’s attitude to weight loss were affected by a lack of access to public transport, affordability of gyms or personal trainers, and specific cultural dietary needs. This paper was conducted in rural New Zealand so may only relate to this specific population; however, this illustrates the importance of acknowledging the unique needs of a community when developing guidelines and resources.

#### Theme 2: Medicalisation of obesity

HCPs in eight papers expressed perceptions around the non-medicalisation of obesity (Brown and Thompson, 2007; Phillips *et al*., 2014; Kirk *et al*., 2014; Blackburn *et al*., 2015; Hayes *et al*., 2017; Brautigam Ewe *et al*., 2021; Aboueid *et al*., 2022; Norman *et al*., 2023). HCPs reported that they do not deal with obesity itself but ‘*reactively*’ treat associated co-morbidities (Hayes *et al*., 2017). One HCP said it was within the ‘*culture*’ of medicine to deal with the ‘*issues*’ rather than the underlying cause (Kirk *et al*., 2014). Other HCPs said they would ‘*never*’ bring up weight unless a patient referred to it themselves (Brown and Thompson, 2007). HCPs felt they were hindering the therapeutic relationship by bringing up the topic of weight unannounced (Blackburn *et al*., 2015). HCPs also demonstrated a tendency towards non-medicalisation of obesity by focusing on weight loss, healthy eating and exercise in an effort to avoid labelling patients instead of using the words ‘*obese*’ and ‘*obesity*’ (Brown and Thompson, 2007). Additionally, two HCPs felt discussing weight was inappropriate due to wider social, psychological and cultural factors affecting a person’s weight, as demonstrated in the paragraphs above. One HCP said obesity should be dealt with ‘*outside the NHS’* (Blackburn *et al*., 2015), and another explained they did not have resources to deal with the multiple factors affecting obesity and, thus, did not raise the topic (Hayes *et al*., 2017).

Alternatively, HCPs in three papers identified positive reasons for raising the topic of weight with an overweight patient, even if they were not unhealthy (Phillips *et al*., 2014; Brautigam Ewe *et al*., 2021; Aboueid *et al*., 2022). A participant explained this could ‘*prevent ill health and complications’* (Phillips *et al*., 2014). One HCP said that most people were ‘*aware that they are overweight and could lose a few pounds’* and so did not find it difficult to approach the topic (Aboueid *et al*., 2022). These statements illustrate agreement with medicalisation of obesity where being overweight with no other co-morbidities is enough to merit weight loss advice (Sadler *et al*., 2009).

#### Theme 3: Organisational factors

Multiple organisational factors affecting HCPs’ ability to deliver weight management care were identified. HCPs expressed a lack of knowledge to give sufficient weight loss advice, a lack of resources available and an absence of multidisciplinary approaches. Lastly, many acknowledged the sensitivity of the topic and stated they did not have time for a thorough conversation surrounding weight. HCPs in three papers expressed they felt there was little focus on obesity management within their organisation (Bornhoeft, 2018; Holmgren *et al*., 2019; Brautigam Ewe *et al*., 2021). The factors identified illustrate this lack of focus in terms of the subthemes: knowledge, resources and time.

#### Knowledge

HCPs illustrated they were unaware of current dietary advice and guidelines, what resources were available or how to raise conversations surrounding weight loss. Seven papers acknowledged HCPs lacked necessary knowledge to approach the topic of weight with patients (Gunther *et al*., 2012; Kirk *et al*., 2014; Blackburn *et al*., 2015; Bornhoeft, 2018; Holmgren *et al*., 2019; Brautigam Ewe *et al*., 2021; Norman *et al*., 2023). HCPs said they did not know *‘how to approach the subject’* (Bornhoeft, 2018) or they did not get ‘*any training*’ surrounding raising the topic of weight (Gunther *et al*., 2012). HCPs illustrated fears of approaching the topic due to the impact it may have on patients. They were aware patients may feel ‘*judged*’ (Blackburn *et al*., 2015) or that raising the topic may create tensions between patient and HCP (Kirk *et al*., 2014). In short, a fear of breaking down the therapeutic relationship presents a barrier to approaching the topic of weight.

Additionally, HCPs felt they lacked the knowledge on weight loss methods and did not know which ‘*diets’* to advise (Bornhoeft, 2018). Practice nurses in Mercer and Tessier’s paper (2001) wanted distance learning, seminars or papers to update their knowledge on nutrition. In contrast, some HCPs were able to confidently raise the topic of weight and act accordingly saying they *‘always’* ask patients if they would like to be weighed (Gunther *et al*., 2012).

Greater experience influenced HCPs’ ability to discuss weight with patients. One paper illustrated nurses could be more sensitive and raise delicate topics through experience, finding it easier to have conversations surrounding weight from experience of talking to patients about lifestyle changes (Holmgren *et al*., 2019). However, nurses from this paper held a master’s degree in Public Health which may have improved their ability to raise sensitive topics. Blackburn’s theoretical domains framework paper (2015) further illustrated how nurses that took part in research surrounding obesity found it easier to raise the topic of weight due to increased confidence (Blackburn *et al*., 2015).

A lack of a standardised procedure for the management of overweight patients further appeared to contribute to difficulties surrounding discussions about weight. One HCP said ‘*guidelines are always changing, I am not sure where to access these guidelines’* (Bornhoeft, 2018) and seven other papers illustrated low awareness of a standardised guideline (Merser and Tessier, 2001; Brown and Thompson, 2007; Ali *et al*., 2008; Blackburn *et al*., 2015, Dunkley *et al*., 2009, Hayes *et al*., 2017; Brautigam Ewe *et al*., 2021). Conversely, Gunther *et al*. (2012) illustrated the benefits of having a standardised guideline for managing obesity ingrained within practice policies. Nine GP practices were included in this paper: one with a localised standardised guideline for managing obesity explained that with the introduction of their guideline they were *‘increasingly aware’* of factors to address and recognised co-morbidities associated with obesity as a *‘parcel’* of factors they were unable to ignore (Gunther *et al*., 2012). In contrast, Brown and Thompson (2007) illustrated that practitioners who were unaware of a standardised guideline found it more difficult to have a conversation surrounding weight. Experience and knowledge, and implementation of a standardised guideline were perceived to be helpful in discussing weight management with patients.

#### Resources

As mentioned above, some HCPs were able to confidently address a patient’s weight. However, one paper suggested HCPs only raise the topic of weight when they have sufficient advice and resources to offer saying there was ‘*no point’* if these elements were absent (Blackburn *et al*., 2015). This further illustrated the frustration felt by HCPs that do not have resources to offer patients looking to lose weight. One HCP said, ‘*I feel constrained in what I can do’* (Gunther *et al*., 2012). Another said they were sceptical about referring patients to a weight management programme as they lacked ‘*hope*’ it would work (Kirk *et al*., 2014). HCPs in three papers mentioned which resources were lacking saying, *‘an obesity clinic or dietitian clinic and group education’* (Ali *et al*., 2008) and a ‘*lifestyle reception’* (Brautigam Ewe *et al*., 2021) were required and explained that a referral was difficult as they had reduced dietary services in certain practice areas in this paper’s sample (Gunther *et al*., 2012).

Input from other professionals was perceived as valuable. For example, one HCP in Bornhoeft’s paper (2017) said a *‘team’* was required adding, ‘*we need a nutritionist, an exercise person, and maybe a psychologist to deal with all the psychological stuff’.* This was further illustrated in Holmgren *et al*. (2019) by an HCP who said ‘*multi-professional collaboration’* was necessary and a dietician *‘would have been worth gold’* (Brautigam Ewe *et al*., 2021). However, this desire for a multidisciplinary approach was contradicted by two papers illustrating a lack of accountability within a practice team for the responsibility of weight management (Mercer and Tessier, 2001; Hayes *et al*., 2017). HCPs said, *‘the PA (Physician Assistant)’* was primarily responsible, while a Physician Assistant from the same paper said*, ‘the physician is almost always responsible’* (Hayes *et al*., 2017) and ‘*We very much leave it to the PN (Practice Nurse)’ (*Mercer and Tessier, 2001).

#### Time

Eight papers reported HCPs did not feel they had time to adequately raise the topic of weight with patients (Merser and Tessier, 2001; Dunkley *et al*., 2009; Gunther *et al*., 2012; Blackburn *et al*., 2015; Bornhoeft, 2018; Holmgren *et al*., 2019; Brautigam Ewe *et al*., 2021; Norman *et al*., 2023). One paper stated HCPs’ contracts included bonuses for seeing as many patients as possible saying ‘*I have to keep my numbers up*’ and ‘*my bonus depends on it’,* implying there is no time to talk about weight (Bornhoeft, 2018). However, these results will only apply to specific healthcare systems where a bonus system is employed and is not relevant to healthcare systems such as the UK. Two papers undertaken in the UK illustrated that a 10-minute consultation was not enough to adequately discuss weight, and HCPs chose to speak about other health promotion factors such as smoking over weight management (Dunkley *et al*., 2009; Blackburn *et al*., 2015). Another paper noted that when the practice was understaffed, routine work such as injections was prioritised over discussions about lifestyle changes (Holmgren *et al*., 2019). HCPs also feared that weight discussions would increase their workload (Dunkley *et al*., 2009). While most papers identified there was a lack of time to address the issue, one paper addressed a need to make time to manage weight, explaining that a designated five-minute time slot to discuss weight was necessary (Rand *et al*., 2017). However, this paper was primarily based around psychological factors contributing to weight gain which although important, may not be relevant to initiating a general conversation about weight loss.

#### Theme 4: Lack of patient knowledge and motivation

In nine papers, HCPs reported a recognition of patient knowledge and motivation as playing a role in weight management (Mercer and Tessier, 2001; Brown and Thompson, 2007; Ali *et al*., 2008; Phillips *et al*., 2014; Bornhoeft, 2018; Rand *et al*., 2017; Holmgren *et al*., 2019; Brautigam Ewe *et al*, 2021; Aboueid *et al*., 2022). HCPs felt that patients required knowledge surrounding healthy diets for them to effectively manage their weight. This was demonstrated by HCPs saying that patients did not understand the ‘*seriousness’* (Bornhoeft, 2018) of being overweight and did not consider it a ‘*problem’* (Ali *et al*., 2008). HCPs also suggested patients who did want advice wished for ‘*easy*’ ways to lose weight and were unaware people rarely comply with ‘*restrictive’* diets (Brown and Thompson, 2007). Therefore, HCPs felt a responsibility to ‘*educate*’ patients to successfully make positive lifestyle changes for weight loss (Bornhoeft, 2018), and that patient ‘*education and compliance’* was a key factor in successful weight loss (Brown and Thompson, 2007).

Some HCPs perceived patients lacking in motivation would be unsuccessful in losing weight. HCPs said motivation was ‘*key*’ (Bornhoeft, 2018) to weight loss and patients require ‘*willpower*’ to choose healthy food (Rand *et al*., 2017). HCPs also illustrated the importance of patients motivating themselves to lose weight rather than the doctor, saying if the patient was ‘*happy’* being overweight then they would not lose weight (Mercer and Tessier, 2001). This further illustrates the need for a collaborative approach between patient, HCP and the multidisciplinary team.

## Discussion

This qualitative systematic review aimed to identify the perceptions of HCPs towards obesity and obesity management, experiences of developing and maintaining a therapeutic relationship when discussing obesity and obesity management with patients, and resources needed to develop a therapeutic relationship with patients who are overweight or obese. In relation to the objectives, this review has identified that HCPs have an extremely broad and widely varied perception of and experiences of managing patients who are overweight or obese. HCPs can find it difficult to have a discussion surrounding weight for fear of offending the patient and, therefore, upsetting the therapeutic relationship whilst also acknowledging that there must be a therapeutic relationship present before engaging in discussions surrounding weight. Whilst some HCPs did not acknowledge the therapeutic relationship, there was a wide understanding that this patient group experiences stigma within society that HCPs did not want to further contribute to. There is a lack of specific training, resources and standardised guidelines available for HCPs that will enable them to discuss weight whilst maintaining the therapeutic relationship.

HCPs acknowledged a range of perceptions regarding weight and weight management in primary care. These perceptions ranged from acknowledging the wider social and psychological factors affecting weight and acknowledging stigma, while other HCPs held a reductionist view of the factors affecting someone’s weight and actively enforced said stigma. Negative perceptions such as these further enforce harmful ideas about people who are overweight, leading to negative physical and psychological consequences for the individual (Ogden, 2014). Not only does this illustrate the conscious or unconscious influence of societal norms in HCPs care, but it also illustrates the need for education and training on the vast array of factors influencing weight. Interestingly, only one paper specifically acknowledged the unique socio-economic factors of their research population, whilst others did not explicitly comment on the effect this may have had on results. Whilst noting the differences in socio-economic factors is positive, this makes the development of a standardised guideline challenging due to the unique needs to each community. However, it would not be feasible or economically viable to develop individualised guidelines and/or resources for each community/area, but it is worth noting that more deprived areas may require more support and guidance from HCPs than affluent areas.

In addition, HCPs acknowledged that their own weight played a role in their ability to provide weight management care. Overweight and obese HCPs were able to empathise with overweight and obese patients and acknowledge the stigma they may experience. They were also able to offer advice and guidance based on their own personal experience of weight loss; however, this method of weight management care may be questioned due to reliance on personal experience as opposed to research evidence.

Some HCPs showed ideas in line with the ‘plus-size’ movement in that they felt some weight management guidelines were extreme and overweight patients can be healthy. It might be argued that while this is beneficial for reducing stigma, this increases the risk of undermining health consequences associated with being overweight (Muttarak, 2018). Additionally, arguments for and against the medicalisation of obesity are potentially linked to the therapeutic relationship, for example, it might be suggested that HCPs would not bring up the topic of weight with an otherwise healthy person for the fear of unnecessarily offending, labelling or stigmatising the individual. It is paramount that the therapeutic relationship exists for effective weight management care, for example, The World Health Organization outlines that education, engagement and empowerment are key to a patient’s weight loss journey (World Health Organization, 2021) Therefore, it might be proposed that there are benefits to identifying and treating individuals who are overweight early to prevent potential co-morbidities that may occur. Further research is required to identify benefits of raising the topic of weight early with patients. Additionally, further research, education and training are required to provide HCPs with the skills to provide weight management care whilst eliminating stigma and maintaining the therapeutic relationship.

Lastly, organisational factors played a large role in HCPs ability to raise the topic of weight. A larger organisational focus on weight management is required as well as an increase in the level of education, training and resources dedicated to weight management care. It could also be argued that facilitating a Multidisciplinary Team (MDT) approach will allow for greater holistic management of weight and assist HCPs in managing physical, social and psychological factors affecting weight. These changes in organisations will not only assist HCPs to provide collaborative, high quality, evidence-based, non-stigmatising weight management care but also reduce the cost and effect of co-morbidities of overweight and obesity on health services across the globe.

The review used a systematic approach to identify relevant evidence, following PRISMA guidelines and assessing the quality of included papers. The papers we identified for inclusion were of sufficiently high quality, illustrating how their data analysis was rigorously conducted and stating how themes or trends were identified and backed up by secondary researchers. The overall high quality of included papers lends cautious support to our synthesised findings and conclusions. However, it must be noted that many papers failed to outline the relationship between participant and researcher, which may contribute to bias in our findings. Additionally, the majority of papers recruited participants using purposive sampling which again is subject to researcher bias (Sharma, 2017).

We acknowledge that there were some limitations to the review. Expanding the inclusion criteria, such as including grey literature and studies which were not in English as well as searching more databases may have allowed for a higher variety of geographical representation (most studies were from North America and the UK), widened the scope of the review and generated more data and insights.

### Implications for practice and/or policy and research

Conversations about weight appear to be subject to various organisational barriers including lack of resources such as time, particularly in the current climate, as many global healthcare systems deal with and recover from the coronavirus pandemic. In addition, the NHS specifically is struggling with long waiting lists, staffing issues and public sector strikes. Managing obesity and changing practice in this climate is extremely challenging. However, arguably managing weight may be an increasingly imminent issue as inequalities within society and within health have widened since the pandemic, thus whilst challenging, public health initiatives are required. The argument could therefore be made that it is important these conversations between HCPs and patients take place to prevent exacerbation of the obesity crisis in the future. Further to this, it is evident that conflicting discourses exist in relation to obesity and that some HCPs hold views which stigmatise people who are overweight. This can directly affect the therapeutic relationship and patient engagement with health services. It is crucial that clear, specific and accurate information is provided to HCPs to challenge these views. Future research should focus upon developing and evaluating cost-effective ways to achieve this – whether through refined clinical guidelines, education packages or other such interventions. To summarise, below are some key action points for how practice, policy and research can move on from this review:

- Further research is required to increase understanding of the influence of societal weight norms on the therapeutic relationship between HCPs and patients in primary care.
- This research and future research should be used to guide the development of resources and training which will facilitate positive weight management conversations.
- Positive research results should be used to develop a standardised guideline to reduce the variability in weight management care provided by HCPs.
- A multidisciplinary approach must be applied to manage the vast range of factors that affect weight.
- Improved therapeutic relationships and reduced stigma between patients and HCPs will lead to increased engagement in weight management and a reduction in overweight/obesity.

## Conclusion

Current research suggests a greater need for organisational approaches to improve education and quantity of resources available for staff to manage patients’ weight in primary care settings. The evidence base shows vast geographical and cultural scope, illustrating the enormity of the problem. The severity of the global obesity pandemic means all measures to enable populations to become a healthy weight must be taken; thus, it is paramount to understand these effects. Tackling this issue against the challenge of resource-constrained healthcare settings in light of COVID-19 requires careful development, evaluation and implementation of cost-effective interventions.
